# Dynamics and Reversibility of the DNA Methylation Landscape of Grapevine Plants (*Vitis vinifera*) Stressed by *In Vitro* Cultivation and Thermotherapy

**DOI:** 10.1371/journal.pone.0126638

**Published:** 2015-05-14

**Authors:** Miroslav Baránek, Jana Čechová, Jana Raddová, Věra Holleinová, Eva Ondrušíková, Miroslav Pidra

**Affiliations:** Mendeleum—Department of Genetics, Horticulture Faculty of Mendel University in Brno, Lednice, Czech Republic; Institute of Crop Sciences, CHINA

## Abstract

There is relatively little information concerning long-term alterations in DNA methylation following exposure of plants to environmental stress. As little is known about the ratio of non-heritable changes in DNA methylation and mitotically-inherited methylation changes, dynamics and reversibility of the DNA methylation states were investigated in grapevine plants (*Vitis vinifera*) stressed by *in vitro* cultivation. It was observed that significant part of induced epigenetic changes could be repeatedly established by exposure to particular planting and stress conditions. However, once stress conditions were discontinued, many methylation changes gradually reverted and plants returned to epigenetic states similar to those of maternal plants. In fact, in the period of one to three years after *in vitro* cultivation it was difficult to distinguish the epigenetic states of somaclones and maternal plants. Forty percent of the observed epigenetic changes disappeared within a year subsequent to termination of stress conditions ending and these probably reflect changes caused by transient and reversible stress-responsive acclimation mechanisms. However, sixty percent of DNA methylation diversity remained after 1 year and probably represents mitotically-inherited epimutations. Sequencing of regions remaining variable between maternal and regenerant plants revealed that 29.3% of sequences corresponded to non-coding regions of grapevine genome. Eight sequences (19.5%) corresponded to previously identified genes and the remaining ones (51.2%) were annotated as “hypothetical proteins” based on their similarity to genes described in other species, including genes likely to undergo methylation changes following exposure to stress (*V*. *vinifera* gypsy-type retrotransposon Gret1, auxin-responsive transcription factor 6-like, SAM-dependent carboxyl methyltransferase).

## Introduction

Very early in the history of plant tissue culture it was observed that clonally propagated plants often exhibit some level of variability, termed somaclonal variation [1]. The occurrence of phenotypic or genetic changes after *in vitro* cultivation depends on a wide range of factors, including the original role of the cultured tissue and the plant’s regeneration systems [2,3]; the strength and duration of stressful conditions may also play a role [4,5,6].

How a single progenitor plant can produce a variety of phenotypic outcomes under the same *in vitro* culture conditions is still far from completely understood, but it is likely to result from various causes. Genetic changes observed in regenerated plants include alterations in chromosome number, point mutations and new insertions of transposable elements [7,8]. Epigenetic variation, even in the absence of phenotypic variation, has been observed many times [9,10,11]. Last but not least, changes in phenotype may reflect effects occurring *in vitro*, such as chimerical segregation or loss of a pathogen [9].

The role of epigenetic factors in the phenomenon of somaclonal variation has often been noted and is highly important [10]. Generally, epigenetic changes can be described as heritable alterations in the expression of the information encoded in DNA, although the primary structure of the DNA itself remains unchanged. The main epigenetic factors influencing gene expression are changes in DNA methylation (or hydroxymethylation), modifications of histones or both [12]. Methods to assess DNA methylation and chromatin modifications have been developed [13,14,15], allowing an accurate evaluation of the epigenetic status. Histone modifications are studied using chromatin immunoprecipitation (ChIP) of associated DNA, followed by amplification of cDNA using the polymerase chain reaction (PCR), or else by the whole genome microarray hybridisation [16,17,18].

Various methods and protocols enable detection of DNA methylation. High performance liquid chromatography (HPLC) or high performance capillary electrophoresis (HPCE) can be used to study global DNA methylation, defined as the relation between methylated deoxycytosines and total deoxycytosines [19]. To study sequence-specific DNA methylation, methods based on bisulphite conversion may be used, as treating DNA with bisulphite converts unmethylated cytosines into uracil but does not change methylated cytosines [20,21]. More recently, a strategy using methylated DNA immunoprecipitation followed by high-throughput sequencing (MeDIP-seq) was described to analyse the DNA methylome [22].

Another principle is employed within the MSAP (Methylation Sensitive Amplified Polymorphism) protocol. MSAP generally makes use of a pair of methylation sensitive restriction enzymes, which recognise the same sequence, but have differential sensitivity to methylation at cytosines within recognised site. Selective PCR amplification followed by comparison of the spectra generated by each enzyme of the isoschizomer pair allows determination of the cytosine methylation status at the restriction site, most frequently CCGG loci.

On the other hand, it is necessary to admit that MSAP also has its limitations. In fact MSAP can investigate only a small proportion of the methylated cytosines in the genome, and is also limited by distinguishable scale of variation in methylation within recognised site (CCGG loci). Recently some another factors were described as a theoretically playing role within process of polymorphic signal origination. For example was described slightly different melting point of methylated and unmethylated loci [23,24] or effect of proteins remaining in DNA isolates on amplificability of methylated or unmethylated loci [25]. But in spite of these circumstances MSAP method is still frequently used as a important source of quality data for quality articles [26,27,28], whereas MSAP has been also widely applied to problems arising from epigenetic changes following *in vitro* cultivation of plants [29,30,31,27].

To date, many problems have been solved by analyses focusing on DNA methylation, but relatively few have included evaluation of the role of time on the epigenetic state of plants. Considerations of time are usually restricted to the duration of *in vitro* cultivation and its effect on the degree of epigenetic change [32,30,33], although dependency of the plant age on general DNA methylation was also observed [34]. Studies focusing directly on observations of the DNA methylation states of the plants in different time periods after exposure to stress are largely absent. Consequently, our previous and recent analyses attempt to fill this gap in the knowledge.

The initial impulse to perform this work arose from the desire to determine whether the unique and long-established properties of grapevines were threatened by *in vitro* techniques used in modern viticulture (for propagation, thermotherapy, somatic embryogenesis etc.). Such changes are particularly undesirable in the case of grapevine clones, which possess unique characteristics resulting from purposeful selection carried out over centuries. In previous studies [35,36], using standard Amplified Fragment Length Polymorphism (AFLP), we showed genetic changes caused by *in vitro* thermotherapy were rare, but we found noticeable changes in DNA methylation, when regenerant and maternal plants were compared using MSAP [37]. More recently, when comparing a regenerant with maternal plants up to 5 years after *in vitro* thermotherapy, we registered a shift of epigenetic state of regenerant plants back to that of the maternal plants [36]. Furthermore, we found our capacity to differentiate clearly between the epigenetic states of maternal and *in vitro* regenerant plants was time-dependent and the critical limit lay somewhere between 6 weeks and 3 years after *in vitro* manipulation.

The DNA methylation state of grapevine plants within this critical period following thermotherapy was compared with that of maternal plants to monitor the effect of time on the frequency of registered DNA methylation changes. This provided important information about dynamics and reversibility of DNA methylation landscape in regenerant plants. Our current and previous results allowed determination of the critical time period during which epigenetic states of maternal and *in vitro* regenerant plants could be distinguished using MSAP. In addition, a number of MSAP amplicons that remained polymorphic between maternal and regenerant plants 1 year after thermotherapy were also sequenced and characterised. This is the first study examining DNA methylation over a long timeframe following after exposure of grapevine plants to stress.

## Materials and Methods

### Plant material

Vines (*V*. *vinifera* L.) with confirmed virus-free status were selected as progenitor plants (one for cv. Müller Thurgau and one for cv. Riesling) and planted in an insect-proof greenhouse in Lednice, Czech Republic. Woody stems from each of these two original 5-year-old progenitor plants were cut into 20 single node pieces in winter 2010, planted into plastic containers with soil and reared as individual plants numbered 1–20. Some cuttings (nos. 8–20 for both cultivars) were inoculated in autumn 2010 with Grapevine fanleaf virus (GFLV). To confirm the success of GFLV inoculation, RT-PCR testing for GFLV presence was performed using standard procedures and primers previously described by Mackenzie et al. [38]. Cuttings which repeatedly showed positive reaction to GFLV tests as well as healthy cuttings (nos. 1–7, not inoculated) were then used repeatedly as sources to establish *in vitro* cultures and *in vitro* thermotherapy in 2011 and 2012.

### *In vitro* cultivation

*In vitro* cultures were established using nodal segments grown on a Murashige and Skoog (MS) [39] medium containing 1.33 μM 6-benzylaminopurine (BA) and 0.57 μM indole-3-acetic acid (IAA; both supplied by Serva). The cultures were maintained at 23°C in a 16/8 h cycle of light and dark. Fluorescent tubes with cool white light were used with the photosynthetic photon flux (PPF) adjusted to 20.2 μmolm^-2^s^-1^. The experimental plants were transferred to fresh medium after 3 weeks and each plant was placed into a separate test tube. Six-week-old cultures were either kept on current medium, or exposed to *in vitro* thermotherapy.

### *In vitro* thermotherapy and transfer to non-sterile conditions

Plants propagated on MS medium were placed in a thermobox with a 16/8 h light/dark cycle and kept at 37°C. Relative humidity and light intensity were set at 80% and 22 μmolm^-2^s^-1^, respectively. After 45 days, apical segments were sampled and placed on fresh MS medium. After both types of treatment (i.e., simple *in vitro* cultivation and *in vitro* thermotherapy), the plants were transferred to MS media containing 0.81 μM 1-naphthalene-acetic acid (NAA; supplied by Serva) to support their further growth and rooting. All rooted plants were simultaneously placed into a peat substrate with added agriperlite. To confirm the success of thermotherapy, RT-PCR tests were performed under conditions by Mackenzie et al. [38].

### Selection of plants for MSAP analysis

**Fig 1:**
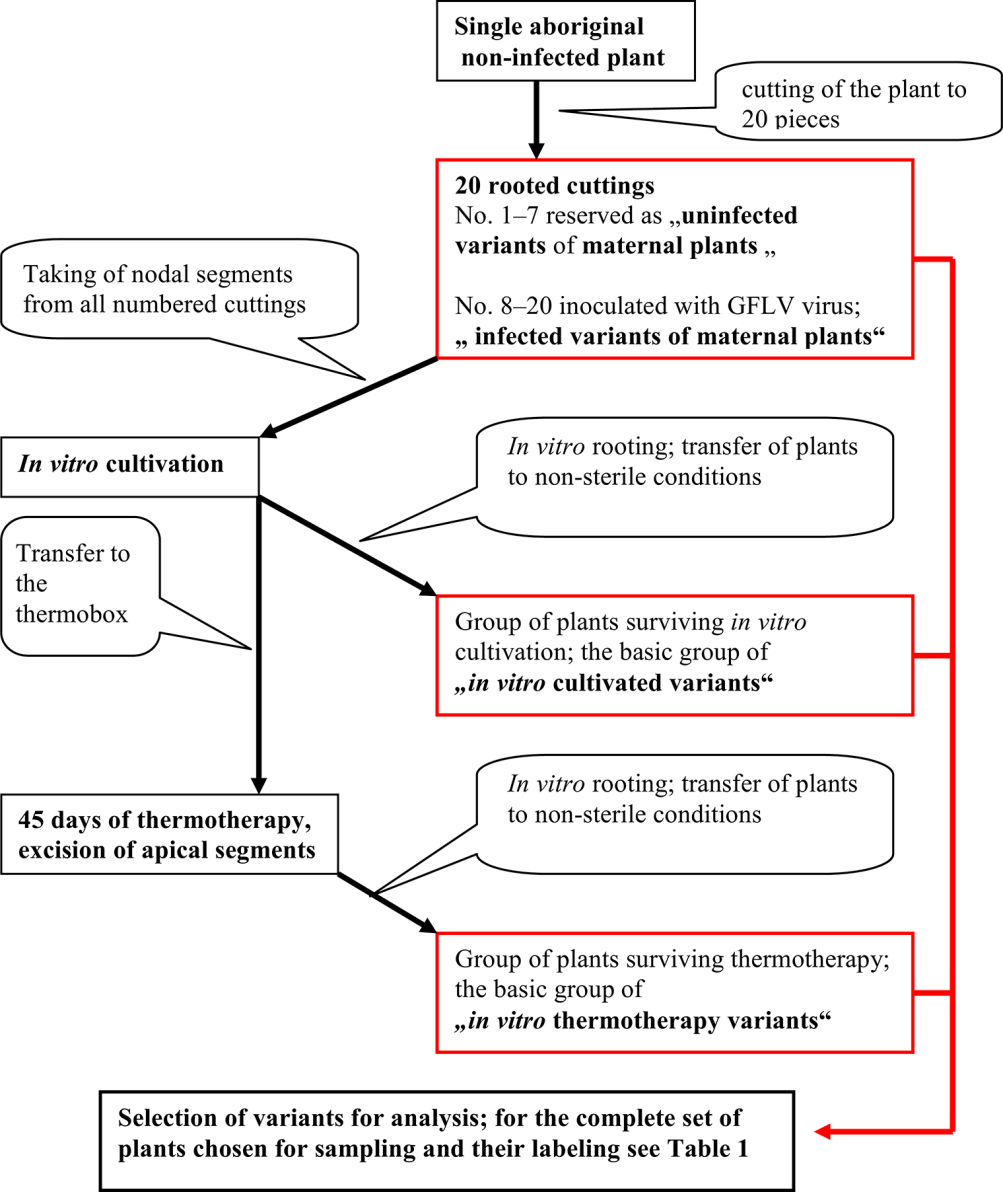
Schema of the preparation of individual variants.

The preparation of plants which went through *in vitro* cultivation and *in vitro* thermotherapy was performed in 2011 and 2012 under conditions described above. The aim was to obtain a series of regenerant plants grown for different periods of time after their exposure to different types of stress conditions (simple *in vitro* cultivation, *in vitro* thermotherapy and virus infection). The entire process of the experiment set-up is described in Fig 1.

Finally, only a complete series of regenerants originating from one known maternal cutting was selected for subsequent work. The final group of variants used in MSAP analysis is shown in Table 1.

**Table 1:** The group of variants analysed by MSAP.

List of maternal plants	List of regenerated plants 1 year after *in vitro* cultivation	List of regenerated plants 1 year after thermotherapy	List of regenerated plants 8 weeks after *in vitro* cultivation	List of regenerated plants 8 weeks after thermotherapy
R-2-M-NI	R-2-IV-NI-(1 year)	R-2-TIV-NI-(1 year)	R-2-IV-NI-(8 weeks)	R-2-TIV-NI-(8 weeks)
R-9-M-I	R-9-IV-I-(1 year)	R-9-TIV-HAI-(1 year)	R-9-IV-I-(8 weeks)	R-9-TIV-HAI-(8 weeks)
MT-1-M-NI	MT-1-IV-NI-(1 year)	MT-1-TIV-NI-(1 year)	MT-1-IV-NI-(8 weeks)	MT-1-TIV-NI-(8 weeks)
MT-8-M-I	MT-8-IV-I-(1 year)	MT-8-TIV-HAI-(1 year)	MT-8-IV-I-(8 weeks)	MT-8-TIV-HAI-(8 weeks)

The system used to identify individual variants in Table 1 is as follows: the cultivar abbreviation (MT = Müller Thurgau or R = Riesling) is followed by identification number of the cutting. Meaning of the suffixes: M-NI = maternal, non-infected plants; M-I = maternal, GFLV-infected plants; IV-NI = *in vitro* cultivated, non-infected plants; IV-I = *in vitro* cultivated, infected plants; TIV-NI = *in vitro* thermotherapy, non-infected plants; and TIV-HAI = *in vitro* thermotherapy, plants healed after GFLV infection. The notes “(1 year)” and “(8 weeks)” indicate the time interval elapsed since the transfer of plants from *in vitro* to non-sterile conditions.

### DNA isolation and sample management

All DNA isolations were performed in one round in September 2012. Tissue samples for DNA isolation were prepared by homogenising three young fully developed leaves from each variant in Table 1. For 10 arbitrarily selected variants their biological replicates were also prepared by DNA isolation from another plant representing the same variant. The pair of biological replicates are distinguished by letter “A” or “B” in the affix of the variant name.

DNA was isolated using a DNeasy Plant mini kit (Qiagen), in accordance with the manufacturer’s instructions. DNA concentration was measured fluorescently using the Quant-iT PicoGreen dsDNA assay kit (Invitrogen) and two samples, each containing 250 ng of DNA, were prepared as input samples for subsequent digestions within the MSAP protocol.

### MSAP analysis and evaluation of results

As a pair of isoschizomers was used *Hpa*II and *Msp*I which recognise tetranuclotide CCGG, but have differential sensitivity to methylation at the inner or outer cytosine. The first sample containing 250 ng of DNA was digested by *Eco*RI and *Msp*I restriction enzymes and the second equivalent sample by *Eco*RI and *Hpa*II restriction enzymes. Subsequent adaptor ligation and pre-amplification reactions, using adaptors and primers designed for *Eco*RI and *Hpa*II/*Msp*I combinations, were performed as in [37]. In the case of selective amplification, three differently labelled *EcoRI*-derived primers, *EcoRI*-ACA (FAM), *EcoRI*-AGC (NED) and *EcoRI*-ACG (JOE), were combined with five *HpaII/MspI*-derived primers, *HpaII/MspI*-TCAA, *HpaII/MspI*-TCAC, *HpaII/MspI*-TCGC, *HpaII/MspI*-GCAT, and *HpaII/MspI*-TACC. This means altogether 15 primer combinations were used. Amplification products were separated electrophoretically using capillary system of ABI PRISM 310 genetic analyser (Applied Biosystems). GeneScan 500 ROX (Applied Biosystems) was used as a size standard and POP 4 polymer (Applied Biosystems) as a medium for fragment separation.

### Data analysis

Accurate interpretation of the spectra was ensured by a detailed independent manual evaluation done by two persons. Moreover, each peak and its intensity was evaluated in the context of the whole group of samples using GeneScan software (Applied Biosystems) to overlap the signals from all samples. Distribution of MSAP amplicons within individual samples (i.e., presence *vs*. absence of a given DNA fragment) was translated into a presence/absence data matrix and typed into a computer file as a binary matrix. Subsequently, MSAP data originating from digestion with *Msp*I and *Hpa*II were put together for each variant and used as a base for calculation of their mutual epigenetic similarity using the Nei and Li/Dice algorithm [40]. Genetic similarity/dissimilarity coefficients were computed using the UPGMA method; corresponding dendrograms were generated using MEGA6 software (http://www.megasoftware.net).

### Sequencing of polymorphic MSAP amplicons

Samples derived from regenerant plants 1 year after *in vitro* thermotherapy were loaded onto 6% non-denaturing polyacrylamide gels together with samples derived from their respective maternal plants. The DNA fragments were visualised using 10,000 × diluted Sybr Gold stain (Invitrogen). After staining, amplicons polymorphic between maternal and *in vitro*-derived variants were identified, excised and placed into micro test tubes designated for use in the FastPrep FP 120 (ThermoSavant) instrument. Gel slices were mashed in the FastPrep by vertical shaking with added beads and DNA fragments were re-amplified using the original MSAP primers (the selective amplification primer was used for the *HpaII/MspI*-derived site and the pre-amplification primer for the *EcoRI*-derived site). Following the re-amplification, primers were removed from the sample using MiniElute PCR Purification Kit (Qiagen). Concentration of the re-amplified DNA was determined using ModulusTM Single Tube Fluorometer (Turner Biosystems) and Quant-iT PicoGreen dsDNA Assay Kit (Invitrogen).

The sequencing reagent mixture for each sample contained 2 μl ABI PRISM BigDye terminator v3.1 (Ready Reaction Cycle Sequencing Kit); 4 μl ABI PRISM BigDye terminator v1.1/3.1 5 × sequencing buffer; 0.4 μl (10 μM) primers corresponding to either the *Eco*RI or *Hpa*II/*Msp*I restriction sites and 10 ng of the re-amplified DNA per 100 bp of the product. Reaction volume 20 μl was finally achieved by adding a necessary amount of H_2_O. All samples were then purified to remove any remaining BigDye reagents using the BigDye Xterminator Purification Kit (Applied Biosystems). The entire procedure was carried out 2–4 times per fragment.

The purified sample (15 μl) was transferred to a 0.5ml test tube and analysed by means of ABI PRISM 310 genetic analyser. Data obtained from these experiments were evaluated using the ‘‘Sequencing analysis” software (Applied Biosystems). A consensus sequence was formed from the replicate sequences obtained for each sample using the CLC Sequence Viewer 6 (CLC Bio) and the consensus sequences were used for database searches within NCBI (National Center for Biotechnology Information) using BLASTN. All of the sequences were also compared with the NCBI database to determine their functional annotations and roles in plant cells, if any.

## Results and Discussion

### General evaluation of computed epigenetic similarity

In all, 30 independent samples representing 20 variants described in Table 1 and biological repeats of 10 arbitrary selected variants were analysed. Subsequently, their 60 MSAP patterns (*Hpa*II and *Msp*I digestions) were evaluated. This lead to a large number of MSAP bands being recognised and scored as present/absent (see S1 and S2 Tables). Among variants derived from grapevine cultivar Müller Thurgau there were evaluated 1854 MSAP bands and among variants derived from Riesling the amount was 2007 MSAP bands. Approximately 64 fragments per primer combination were generated on average for both the cultivars for each primer combination.

Mutual coefficients of similarity calculated on the base of MSAP data from jointed *Msp*I and *Hpa*II digestions are summarised in S3 and S4 Tables. Arithmetic means were 0.8329 for Müller Thurgau-derived variants and 0.7782 for Riesling-derived variants. The highest similarity coefficients across the range of the computed values were, as expected, for the biological repeats, where the mean values were 0.986 and 0.984 for the Müller Thurgau and Riesling cultivars respectively. Excluding repeats, values ranged from 0.70578 (MT-2-TIV-NI-8weeks-B *vs*. MT-1-IV-NI-1year-B) to 0.9571 (MT-1-M-NI *vs*. MT-2-M-NI) within the Müller Thurgau-derived variants and from 0.6453 (R-9-TIV-HAI-8weeks *vs*. R-9-IV-NI-1-year-A) to 0.9652 (R-9-IV-I-1year-A *vs*. R-9-TIV-1year) within the Riesling-derived variants.

### Distribution of variants within a dendrogram

**Fig 2:**
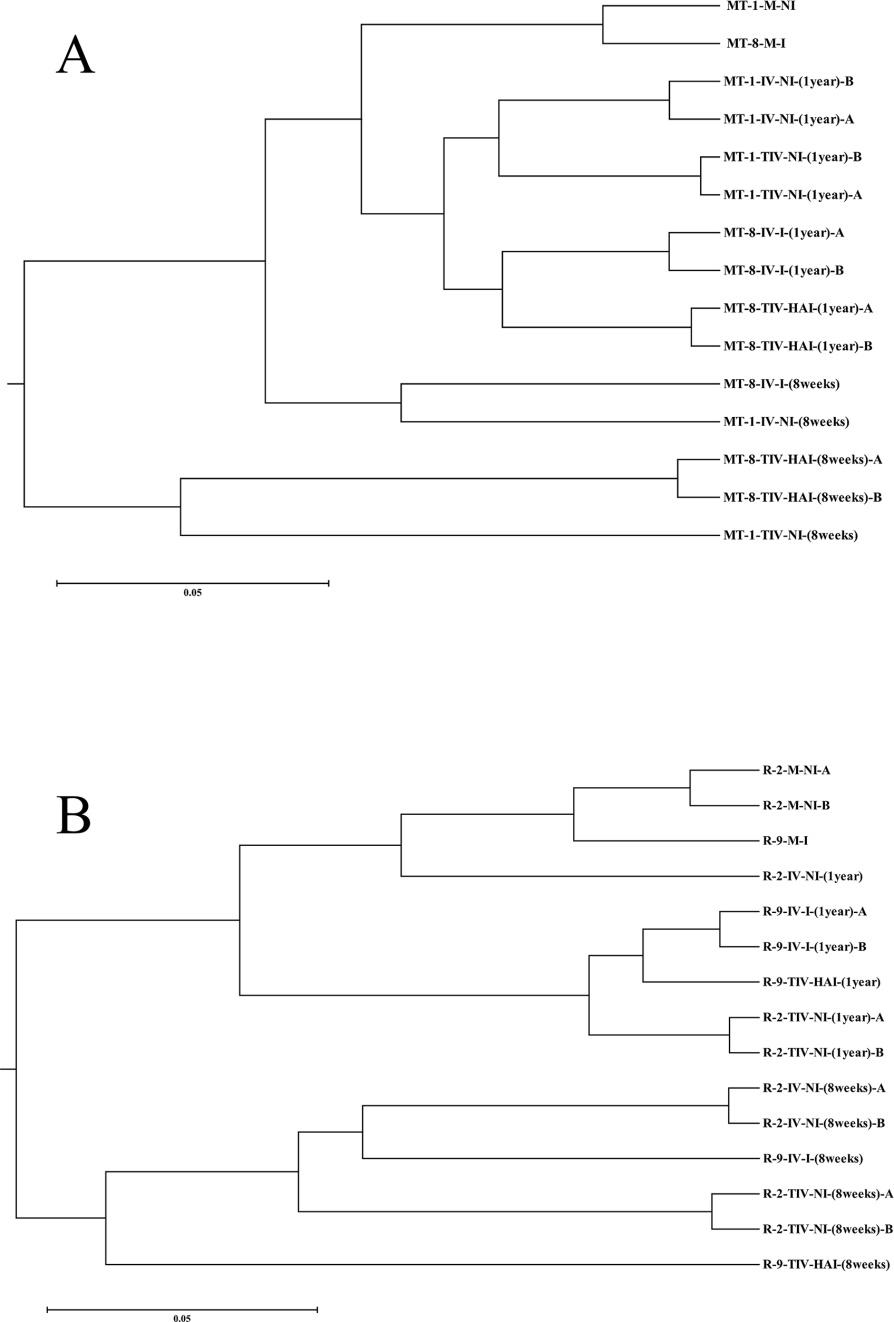
Dendrograms of epigenetic similarity between individuals with different period after stress. (A) Degree of epigenetic changes within variants derived from Müller Thurgau show strong influence of the time which elapsed from the plants's exposure to stress to its testing. Once stress conditions were discontinued, many methylation changes gradually reverted and plants returned to epigenetic states similar to those of maternal plants. Furthermore, it is apparent that epigenetic states in variants after *in vitro* thermotherapy were regularly more different from states in maternal plants than epigenetic states in variants after standard *in vitro* cultivation. (B) Degree and development of epigenetic changes within variants derived from Riesling cultivar. As visible, it is possible to observe the same regularities as for group of Müller Thurgau variants in the Fig 2A. The system used to identify analysed accessions is described in Table 1, biological repeats are recognisable by letters “A” or “B” at the end of the names of respective accessions.

Dendrograms constructed using the computed MSAP spectra similarities clearly showed that biological repeats of the same variants (marked with A and B suffixes) achieved a high degree of similarity (see Fig 2). Confirmation of this very low internal variability within biological replicates was a critical prerequisite for subsequent credible evaluation of the whole group of variants.

Within the used experimental design it is possible to recognise three main factors theoretically playing role in establishing of individual epigenetic states. These factors are *in vitro* stress, stress induced by virus infection and the role of time elapsed since exposure to stress. As visible from dendograms in Fig 2 as well as in S5 Table, the role of virus infection plays negligible role in establishing of individual DNA methylation states, which was also indicated in previous experiments [35, 37]. The reasons are still open with a possible explanation being the confirmed mild character of the applied GFLV isolate, which did not show any usual symptoms many years after infections.

On the other hand, both the dendrograms revealed that DNA methylation states in variants after *in vitro* cultivation are distinctly different from states in maternal plants. This observation is in accordance with other sources, where DNA methylation is described as dynamic process reflecting actual necessities for plant growth and development under prevailing conditions [26]. Moreover, plant variants after *in vitro* thermotherapy show higher degree of DNA methylation differences than variants after standard *in vitro* cultivation if they are compared with states in maternal plants. This confirms a previous observation that healing of plants by *in vitro* thermotherapy induces a higher degree of epigenetic variation than simple *in vitro* culture of nodal segments [37]. This may arise from the effects of subsequently described factors and/or the combination of both. It was reported that plants under severe nutrient and water stress did not exhibit the same DNA methylation changes, as seen after tissue culture and it was, therefore, determined that tissue culture is a unique form of stress, possibly due to addition of plant growth regulators to tissue culture media [41]. Furthermore, in accordance with statements formulated by Gao et al. [42] it is possible to hypothesize that for our thermotherapy variants the stress of higher temperatures induced an array of methylation changes other than those associated with *in vitro* cultivation.

Another explanation arises from the fact that in standard *in vitro* cultivation different tissues are manipulated than those treated by *in vitro* thermotherapy. Apical segments including meristems (used within thermotherapy) and shoot nodes (used within *in vitro* cultivation) probably have different requirements for the regulation of plant development and organ and cell differentiation. As described in recent past, just DNA methylation plays an important role in regulating these processes [43,44,45,11].

As mentioned above, the role of time on DNA methylation drift is generally underestimated and appropriate information are quite rare. Recently Machczyńska et al. [27] published their results regarding DNA methylation content within different kinds of in vitro cultures of triticale and their progeny. They demonstrated that in vitro cultivation initially induced a decrease of the DNA methylation of the regenerants, whereas decrease in DNA methylation proceeded up to the first/second successive generations followed by the beginning of initial DNA methylation content reestablishment.

Regarding presented results, general clustering pattern in the dendrograms (Fig 2) also clearly demonstrated the importance of time elapsing after *in vitro* planting on the epigenetic drift of regenerant plants back to a state resembling corresponding maternal plants. More precisely, regenerant plants tested soon (8 weeks) after exposure to *in vitro* conditions showed significantly greater DNA methylation changes than those tested after a longer period (1 year). A year after *in vitro* culture, the epigenetic state was more similar to that of maternal plants, although the two groups could usually still be distinguished. In our previous study [36], we observed regenerant plants forming conjoined clusters with maternal plants 5, 4 and 3 years after *in vitro* themotherapy and epigenetic states of these old regenerant plants merged with those of the maternal plants. When our current and previous results are considered together, it appears that epigenetic states of stressed regenerant and maternal plants become undistinguishable somewhere in the period of 1 to 3 years following the *in vitro* cultivation of the former. Despite an initial disruption of the DNA methylation state in regenerant plants, majority of the induced variation in DNA methylation slowly returns to its original state. This is a reassuring observation for commercial cultivators of grapevines, who would prefer the character of their unique clones not to be altered by *in vitro* thermotherapy.

### Interpretation of observed DNA methylation changes—Are they rather reversible or stable?

Stressing of plants by biotic or abiotic factors induces changes in the activity of different regulators such as chromatin remodelling factors and DNA methylation or demethylation apparatus, both of which are likely to be directed by small RNAs [46]. Parts of these induced alterations are non-heritable changes, caused by transient and reversible stress-responsive gene regulation, allowing plants to acclimate to stressful conditions. Over recent years, however, many examples of stress-induced epigenetic changes that are mitotically or even meiotically heritable have been described [47,48,49].

Despite such reports of stable epigenetic changes, very little is known about the ratio of stable, heritable epigenetic modifications to reversible DNA modifications. In this context, a unique view is provided by further (statistical) processing of the obtained results, where data from individuals with the same attributes (time elapsed since exposure to stress and the nature of conditions during *in vitro* cultivation) were collated and DNA methylation similarities within and between the groups were compared (Table 2).

**Table 2:** Comparison of average mutual epigenetic similarities between groups represented by individuals with the same attributes (time elapsed since exposure to stress and the nature of conditions during *in vitro* cultivation).

State of plant material	Maternal plants	Standard *in vitro* cultivation/ 1 year after	*In vitro* thermotherapy/ 1 year after	Standard *in vitro* cultivation/ 8 weeks after	*In vitro* thermotherapy 8 weeks after
Maternal plants	MT = 0.9571; *R = 0*.*9504*				
Standard *in vitro* cultivation/ 1 year after	MT = 0.8861; *R = 0*.*8237*	MT = 0.9377; *R = 0*.*9282*			
*In vitro* thermotherapy/ 1 year after	MT = 0.8686; *R = 0*.*8128*	MT = 0.9030; *R = 0*.*8987*	MT = 0.9338; *R = 0*.*9485*		
Standard *in vitro* cultivation/ 8 weeks after	MT = 0.8444; *R = 0*.*7430*	MT = 0.8405; *R = 0*.*7272*	MT = 0.8229; *R = 0*.*7084*	MT = 0.8828; *R = 0*.*8983*	
*In vitro* thermotherapy/ 8 weeks after	MT = 0.7389; *R = 0*.*7340*	MT = 0.7322; *R = 0*.*7205*	MT = 0.7318; *R = 0*.*7157*	MT = 0.7990; *R = 0*.*8038*	MT = 0.8627; *R = 0*.*8381*

The first column clearly shows a decreasing tendency of similarity of DNA methylation landscape, when compared with state in maternal plants. It is also noticeable that the most similar DNA methylation states for each group of variants were usually recorded when individuals from the same group were compared (i.e., values on the diagonal of Table 2).

Regarding ratio of heritable to reversible modifications, very interesting results were obtained from the data in the first column of Table 2. When the coefficients of similarity were re-computed using algorithm of Nei and Li [40], it was possible to extract information about the ratio of monomorphic and polymorphic signals between individual groups of variants. It was found that 40% of the changes seen, when plants were examined immediately after *in vitro* cultivation, had disappeared after 1 year. This figure probably represents the proportion of DNA methylation changes caused by transient and reversible stress-responsive acclimation mechanisms. The remaining 60% of DNA methylation changes persisted also 1 year after *in vitro* cultivation and that is why this figure probably represents epigenetic mitotically-inherited epimutations. Further information concerning this group of mitotically inherited DNA methylation changes was obtained by sequencing of this kind of amplicons (see below).

What was also noticeable, was that the most similar epigenetic states for each group of variants were usually recorded in cases, where individuals from the same group were compared (i.e., values on the diagonal of Table 2). Such an observation suggests that DNA methylation changes did not occur randomly, but exposure of plants to the same stress conditions established similar DNA methylation landscapes. Smulders et al. [50] noted likewise that the same aberration occurred very frequently in a population of plants generated *in vitro*, and it could be reproduced, when the same conditions were imposed during production of another population. Law et al. [51] observed rapid and reversible changes in both DNA methylation and histone modifications in a 15 day period after establishment of a potato cell suspension. Peredo et al. [52] also used molecular techniques to evaluate hop cultures and found nearly 30% of registered epigenetic changes were shared by all *in vitro* plants.

Thus, the same stress conditions resulted in the establishment of similar DNA methylation landscapes. Moreover, the data presented in Table 2 imply that also reversion of the variant epigenetic state to one resembling the maternal plant (see Fig 2) has a similar epigenetic scenario; for example, variants with the same treatment history over the period of one year remain most similar epigenetically, when compared with other variants. This means there must remain enough somatically inherited epigenetic changes to discriminate these regenerants from the epigenetic states of their maternal plant.

Such results suggested the DNA methylation landscapes of individuals treated in similar ways were highly likely to resemble each other. On the other hand, certain proportion of DNA methylation changes did not occur repeatedly in individuals with the same history, indicated by the reduction in the values along the diagonal in Table 2. Such changes were, thus, either generated arbitrarily or a result of another, unknown factor. The lowest degree of similarity in DNA methylation profiles was observed repeatedly in the group of variants 8 weeks after *in vitro* thermotherapy ended. This result indicated that the proportion of arbitrary or uninterpretable changes was highest during the period soon after exposure to stress and also increased with the strength of the stress conditions. In summary, DNA methylation changes within individuals were significantly more frequent soon after exposure to stress or under conditions of strong stressing, when compared to individuals experiencing other conditions, and they were also significantly more chaotic under such circumstances.

The results presented here indicate the plant epigenome is highly plastic, allowing plants to react fast and effectively to stress. A significant proportion of DNA methylation changes was repeatedly induced by exposure to a particular set of planting and stress conditions. Other changes were not repeatable and their causation remains unknown, although the incidence of these changes increased with the strength of the stress and was inversely proportional to the time elapsed since exposure to stressful conditions. After removal of a given set of stressful conditions, individuals began to reverse the changes in their epigenetic state and, eventually, achieved states resembling maternal/non-stressed plants. It remains questionable, if these reversible DNA methylation changes would be best described as quick or slow; however, our recent and previous results indicate that the interval, over which the DNA methylation landscape of plants cultivated *in vitro* becomes indistinguishable from non-stressed controls, lies somewhere between 1 and 3 years.

### Sequencing and database comparisons of fragments with altered methylation

To gain insight into the nature of the fragments containing DNA methylation changes, 58 PCR products, found to be polymorphic between maternal and 1-year-old regenerant plants, were selected for sequencing. There are two explanations for such occurrence of polymorphisms. The first one: the sequences may belong to a DNA methylation hot spot and the observed polymorphism reflects an arbitrary and momentary difference between the samples. The second, and based on these results probably more relevant, explanation is the polymorphic products correspond to stable changes in DNA methylation remaining 1 year after *in vi*tro cultivation. The second explanation is supported by recent findings that the experience of stress encoded by long-term alternations in the epigenetic landscape is seen not only in plants propagated vegetatively, but also in experiments where epigenetic changes were transmitted to progeny [49,53].

From the 58 polymorphic amplicons initially selected, 41 were sequenced successfully (see S1 File) and compared with sequences in public databases using the BLASTN facility of the NCBI website (http://blast.ncbi.nlm.nih.gov/Blast.cgi; conducted on April 2014). The availability of the fully sequenced grapevine genome [54] was a major advantage in this analysis. BLASTN analysis indicated that 12 sequences (29.3%) corresponded to non-coding regions of the grapevine genome and 8 sequences (19.5%) corresponded to previously described genes within grapevine genome. The 21 remaining sequences (51.2%) were highly similar to grapevine whole genome sequenced contigs and simultaneously annotated as “predicted protein” because of their similarity to genes described in other plant species. Some of the identified coding sequences corresponded to genes in which changes to DNA methylationin plants exposed to stress conditions is rather probable.

One of the most evident examples of such correspondence was identity between the Sequence No. 14 in S1 File and part of the gene for *V*. *vinifera* gypsy-type retrotransposon Gret1 DNA (NCBI gene symbol: AB111101.1). The role of retrotransposon in the stress reaction of plants is well-known [55,56,57]. Other sequences showing changes to DNA methylation in this study matched *auxin-responsive transcription factor 6-like* (NCBI gene symbol: LOC100242923), *salicylate carboxymethyltransferase* (NCBI gene symbol: LOC100241069), *PHENYLALANINE AMMONIA-LYASE-LIKE* (NCBI gene symbol: LOC100255939) and also rDNA (see respective Sequences No. 6, 21, 22 in S1 File).

A change in DNA methylation in a gene encoding an auxin-responsive transcription factor is not unexpected in plants developing in tissue culture media. As concerns *salicylate carboxymethyltransferase*, this enzyme show similarity with *S-ADENOSYL-L-METHIONINE (SAM)-DEPENDENT METHYL-TRANSFERASES*, which are crucial for epigenetic mechanisms. They utilise the ubiquitous methyl donor SAM as a cofactor to methylate proteins, small molecules, lipids and nucleic acids, and, thus, mediate numerous biological processes, such as protein trafficking and sorting, signal transduction, biosynthesis, metabolism, and gene expression [58]. Regarding *PHENYLALANINE AMMONIA-LYASE*, this enzyme work as the first step in the phenylpropanoid pathway and is therefore involved in the biosynthesis of the crucial plant polyphenolic compounds as flavonoids, phenylpropanoids or lignins. It was shown that stilben synthases as a genes producing resveratrol—an important metabolite within phenylpropanoid pathway for *Vitis* species—are also regulated via different cytosine methylation in protein-coding regions [59]. In the case of polymorphic sequences originating from rDNA coding regions, hypomethylation of particular rDNA gene families was initiated as early as 2 weeks after callus induction and stably maintained over at least 2 years of cultivation *in vitro* [47].

Another interesting observation we made was that some of the sequences showing polymorphic methylation matched genes containing an F-box domain (see Sequences No. 5, 27, 33, 35 in S1 File). F-box domains play various roles in plant genomes with some F-box proteins functioning as a phytohormone or light receptors [60]. This means F-box domains regulate reaction of the plants to conditions, which are significantly changed by *in vitro* cultivation and, therefore, it is likely that DNA methylation plays a significant role in such regulation.

## Conclusions

In this work we have shown the dynamics and reversibility of DNA methylation landscape found in plants stressed by *in vitro* cultivation. It was determined that a significant fraction of DNA methylation changes could be induced repeatedly by particular combinations of growth and stress conditions, reflected in the observation that individuals given similar treatments showed a significant degree of epigenetic similarity. Other methylation changes did not occur repeatedly in individual variants and their causation remains unknown, although the abundance of such changes increased with the strength of the stress and proximity in time to the end of stress conditions.

Furthermore, we can conclude that the intensity of registered DNA methylation changes, when compared with maternal plants, was strongly influenced by the time which elapsed between the plants’ exposure to stress and the testing, while the epigenetic state of regenerant plants eventually returned to a state similar to their progenitor. From a practical point of view, this information is reassuring to growers of grapevines, as these results imply that superior properties of favourable clones would not be significantly affected by *in vitro* thermotherapy to remove viruses.

Sequencing of DNA regions that remained polymorphic between the regenerant 1 year after *in vitro* cultivation and the maternal plants identified few genes, for which changes in DNA methylation induced by the stressful conditions of *in vitro* cultivation are clearly understandable.

## Supporting Information

S1 File Sequences of polymorphic amplicons. (DOCX)

S1 Table Evaluation of MSAP spectra obtained for Müller Thurgau variants. (XLS)

S2 Table Evaluation of MSAP spectra obtained for Riesling variants. (XLS)

S3 Table Mutual coefficients of MSAP similarity between Müller Thurgau variants. (XLSX)

S4 Table Mutual coefficients of MSAP similarity between Riesling variants. (XLSX)

S5 Table Impact of individual stressing factors on intensity of DNA methylation changes. (DOCX)
