# Supplementary material for: Dynamics and Reversibility of the DNA Methylation Landscape of Grapevine Plants (Vitis vinifera) Stressed by In Vitro Cultivation and Thermotherapy
Source: PLoS One. 2015 May 14;10(5):e0126638. doi: 10.1371/journal.pone.0126638 (PMC4431845; doi:10.1371/journal.pone.0126638)
Supplement: S1 File — (DOCX) [file pone.0126638.s001.docx]

**S1 File. Sequences of polymorphic amplicons**

Sequence No. 1

CGCCATGGCCATGGCGACTCTCTCTCTCTTTGCCTCCACAGATCTAAGAACTATGGCGTGGAATAGTTTAGATTAAGGGCGGCC

Total lenght is 84 nucleotides. BLASTn analysis within NCBI database reveal similarity with:

a)Vitis vinifera contig VV78X224397.26, whole genome shotgun sequence

b) PREDICTED: Vitis vinifera ER membrane protein complex subunit 1 (LOC100264673), mRNA

Sequence No. 2

AATTCACGGGCATAGTGACCAAACTGGGGAAGAATGTGAAGAAATTCAAATTGGGTGACAAAGTTGGGGTGGGGGTGATGGTTGGGTCCTGCAGGTCGTGTGAGAGTTGCAAGCAGGGCTTGGAGAATTACTGCCCCAAAATGATGTTCGCTTACAACTCCATATATCATGATGGCACCAAGAACTATGGTGGTTACTCTAATATGGTGGTGGTTGATCAGCACTTTGTTCTTCGCTTCCCTGATAACCTACCCTTGGC

Total lenght is 259 nucleotides. BLASTn analysis within NCBI database reveal similarity with:

a) Vitis vinifera contig VV78X222566.49, whole genome shotgun sequence

b) PREDICTED: Vitis vinifera probable cinnamyl alcohol dehydrogenase 9 (LOC100250644), mRNA

Sequence No. 3

TTGAAGTCGGCTCTCAAATCAGTTGCCATGTCGTCCTCCACTTCTGGCTCGTTTATAATGGTGAGGGCTATAGGCTCTGGTTCGAGCTTGAAAAAGCAAG

Total lenght is 100 nucleotides. BLASTn analysis within NCBI database reveal similarity with:

a) Vitis vinifera contig VV78X145267.16, whole genome shotgun sequence

b) Vitis vinifera, whole genome shotgun sequence, contig VV78X224772.12, clone ENTAV 115

Sequence No. 4

AATGATTCCTTCAGTTCACGCCCTTGGCAAAAGACACCAGTCTTTACTCTTTACTGCGGTGATTATCGCTTTGACTTACGCTTCCTCACTTGCCTGCATTNAGCTCGGCTGAACAGGNACGCAATCAGTACGCAGTCAA

Total lenght is 139 nucleotides. BLASTn analysis within NCBI database reveal similarity with:

a)Vitis vinifera contig VV78X048242.8, whole genome shotgun sequence

b)PREDICTED: Vitis vinifera putative disease resistance RPP13-like protein 1 (LOC104881345), mRNA

Sequence No. 5

TGTGGACGCTTGCCGTGTGACATGGTCTTGACGCCCTGTGAATT

Total lenght is 44 nucleotides. BLASTn analysis within NCBI database reveal similarity with:

a)Vitis vinifera contig VV78X119259.5, whole genome shotgun sequence

b)PREDICTED: Vitis vinifera putative F-box/LRR-repeat protein At5g02700 (LOC104880213), mRNA

Sequence No. 6

GGAACGGGCTTGGCAGAATCAGCGGTGAAAGAAGACCCTGTTGAGCTTGACTCTAAGTCCGACTTTGTGAAATGACTTGAGAGGTGTAGGATAAGTGGGAGCC

Total lenght is 103 nucleotides. BLASTn analysis within NCBI database reveal similarity with:

a)Vitis vinifera contig VV78X250287.5, whole genome shotgun sequence

b) Vitis vinifera 26S ribosomal RNA gene, partial sequence

Sequence No. 7

AGCCCTGATTTCACTTCTTGAGTTTACACTTTATTCCGTTATGGCCTCAGTGACGACTAATTGGTCTCCGAGCTCGTTCCAGCTCCGTTTCTCCTTCCAATGCAGAAGATCTCCTGCGGTTTTTGTACGGACGCACGTTCGCAAGCTTGATCGTCAGGTTCGCGTGTTGTCCAT

Total lenght is 174 nucleotides. BLASTn analysis within NCBI database reveal similarity with:

a)Vitis vinifera, whole genome shotgun sequence, contig VV78X001441.10, clone ENTAV 115

b)PREDICTED: Vitis vinifera uncharacterized LOC100264485 (LOC100264485), transcript variant X1, mRNA

Sequence No. 8

GAAAGTGCATCCAAAACTTCATGCTCTAGAGCATCAAATGTATTGAAAATGAGGGCAGAAGCCTTGCGAGCTCTTTCAGCCTCCCGCAATGGAAATTCCAGCATAATTTCATTTGGGTCCGTAGTTCGAATGAAGCTTGGGATATCCCTCAAACGGATACCTTTCATACCCG

Total lenght is 172 nucleotides. BLASTn analysis within NCBI database reveal similarity with:

a)Vitis vinifera contig VV78X118531.9, whole genome shotgun sequence

b)PREDICTED: Vitis vinifera 7-deoxyloganetin glucosyltransferase (LOC100243675), mRNA

Sequence No. 9

CTTAGTGGTTTACCATTTGGAGCCGGTAGTGATTGAAAGAAGGAAAAAATAAGACTGGGA AATTGAATCC

Total lenght is 70 nucleotides. BLASTn analysis within NCBI database reveal similarity with:

a)Vitis vinifera contig VV78X041419.2, whole genome shotgun sequence

b)Vitis vinifera contig VV78X263371.5, whole genome shotgun sequence

Sequence No. 10

AATACCGCATTTAGAAGCACATTTACTCCGCAATTTAGACAAAAATGGAATTGTGATACTGGGATCTTGGGTAGAGACGGGCGATATTTTAGTAGGTAAATTAACGCCTCAGATGGCG AAAGAATCATCGTATGCCC

Total lenght is 137 nucleotides. BLASTn analysis within NCBI database reveal similarity with:

a) Vitis vinifera, whole genome shotgun sequence, contig VV78X088483.4, clone ENTAV 115

b) Vitis vinifera subsp. caucasica chloroplast DNA, complete genome, cultivar: Meskhuri Mtsvane

Sequence No. 11

GTCTTCGACATGAAAAGACGATGTCCTAACCACTGGACGAAAGGGACCAAAAAGCCTGATCTTCGACCACCCTAAGCTCCGAGAATAGAAGTGGTTCGTTTTGTTTCTATACGCAATTCAAGATTTCGTTTGTGTTCATGTTGGCGATTTCTGATGTGAATT

Total lenght is 162 nucleotides. BLASTn analysis within NCBI database reveal similarity with:

a)Vitis vinifera strain PN40024 mitochondrion, partial genome

b)Vitis vinifera complete mitochondrial genome, cultivar Pinot noir clone ENTAV115

Sequence No. 12

CTGAGGCTGATCTGGTGGGAAATAGGGTGCGAGTACACACTCTTGCGTACACTTTTGCCG

Total lenght is 60 nucleotides. BLASTn analysis within NCBI database reveal similarity with:

b) Vitis vinifera, whole genome shotgun sequence, contig VV78X152740.2, clone ENTAV 115

a) PREDICTED: Vitis vinifera LOB domain-containing protein 36-like (LOC100246034), transcript variant X5, misc_RNA

Sequence No.13

AGTTACAATCCTCACCGCGTAATAGCGCACCTACCCGTCTTCATCGACGTTGTTTTTTGACCGGAAGACCGGGAGCTAACTATCGATACT

Total lenght is 90 nucleotides. BLASTn analysis within NCBI database reveal similarity with:

a) Vitis vinifera contig VV78X127426.2, whole genome shotgun sequence

b) PREDICTED: Vitis vinifera prostatic spermine-binding protein (LOC100261976), transcript variant X3, mRNA

Sequence No.14

TGCATATGCCATGCATGTGTCCAAGTGGATGTCAAGTTCTCATTTGGTTTACCG

Total lenght is 54 nucleotides. BLASTn analysis within NCBI database reveal similarity with:

a)Vitis vinifera contig VV78X191068.8, whole genome shotgun sequence

b)Vitis vinifera gypsy-type retrotransposon Gret1 DNA and VvmybA1 gene for myb-related transcription factor, complete cds, cultivar: Ruby Okuyama

Sequence No.15

CTGTAGATAGGAGCTCATCATTATATTCCAAGACACAGTATCTTTCTCGGCCATCAGACCAAACAACTTCTCTGCATATCCAAAGCACCTGCATTTCACATAATAATCAAGGATAGAGTTGTTCAACACAGCGTCTAAATCAAGCCCATTCCTCAGAATCCAACCATGAATT

Total lenght is 172 nucleotides. BLASTn analysis within NCBI database reveal similarity with:

a)Vitis vinifera contig VV78X154288.18, whole genome shotgun sequence

b)PREDICTED: Vitis vinifera putative pentatricopeptide repeat-containing protein At3g23330 (LOC100265089), mRNA

Sequence No.16

GACCCAACTTCCATTTTTTCAAAATCTTTGAAAATAATTCTTAATAAAAATTCTTGATTCAAAATCCCAATTTGAAATTTTGAAATGCATAAAGAAATGCTTAAAATGTCATTATGCATG AATTGGTTGC

Total lenght is 130 nucleotides. BLASTn analysis within NCBI database reveal similarity with:

a)Vitis vinifera contig VV78X061745.6, whole genome shotgun sequence

b)Vitis vinifera contig VV78X160004.6, whole genome shotgun sequence

Sequence No. 17

GTCAAACTGGGCTTCCACAAGACCATACTTGTCAAGAATCCAAAACAGACCCATTGCTTTGTTCAGGAGGCATCTTTGCTGATGATAGCAGCCTTGGGCTGGCAGGCATCAAATGGGTAATGGAATCTCTTGTTGAAAATTTTGTATTGGTTGTTCTATGTATGACATAGAATTGCTGATTTGATTTATTTTTTAATGGCTGA

Total lenght is 203 nucleotides. BLASTn analysis within NCBI database reveal similarity with:

a)Vitis vinifera contig VV79X007836.2, whole genome shotgun sequence

b)PREDICTED: Vitis vinifera TSL-kinase interacting protein 1 (LOC100259470), transcript variant X3, mRNA

Sequence No.18

CGGTCAATCAAATAAATAACGAGAAATTAACGTATTGTTGCTCCGTCCATTAGATCCTTCTCTGAAGCTTTTGAGAGGCGAGTTGTTCTTTCTCCTCTTCYATTCCGTTGATTCAGTTGACTTCAATTCAGATCTCTMTCGATCTCAAGTGCTATATCCGCTGATCTCCCCATGCTGATCGGTTGAATT

Total lenght is 189 nucleotides. BLASTn analysis within NCBI database reveal similarity with:

a)Vitis vinifera contig VV78X224919.2, whole genome shotgun sequence

b)PREDICTED: Vitis vinifera ADP-ribosylation factor-like (LOC100245580), transcript variant X1, mRNA

Sequence No.19

GGGACTACAACTTCTCCGAGAAATATGCTGTTAAAAGCCACAGCAGCTATGTGCAGTGACTGAGTAGTTGGGATTTTCGGATAGTAATCATCAGATGCCCGCGTAATTGCACTTCTTCTTGCACTATAATATGATGTAAAGCAGAGTTATGGTTTGG

Total lenght is 157 nucleotides. BLASTn analysis within NCBI database reveal similarity with:

a)Vitis vinifera contig VV78X258854.3, whole genome shotgun sequence

b)PREDICTED: Vitis vinifera probable galactinol--sucrose galactosyltransferase 2 (LOC100251454), mRNA

Sequence No.20

TGCTGGCTACTTGTTTCTTCGTCCGCAGGCATCGCATAAGGCGACAACGACCTAGAGTTTCTCGTGTCCG

Total lenght is 70 nucleotides. BLASTn analysis within NCBI database reveal similarity with:

a) Vitis vinifera contig VV78X211273.4, whole genome shotgun sequence

b) PREDICTED: Vitis vinifera receptor homology region, transmembrane domain- and RING domain-containing protein 2-like (LOC100246359), mRNA

Sequence No.21

AATTCAGCAGCTACCATGAACTGCGTGGTGAGCTTGCTCGCATGTTTGGCCTTGAAGGCCAGTTGGAGGACCCTCGGAGATCAGGCTGGCAGCTTGTATTTGTTGACCG

Total lenght is 109 nucleotides. BLASTn analysis within NCBI database reveal similarity with:

a)Vitis vinifera, whole genome shotgun sequence, contig VV78X073333.6, clone ENTAV 115

b)PREDICTED: Vitis vinifera auxin response factor 6 (LOC100242923), mRNA

Sequence No.22

ACCATTCTTCCTCCCTCAATCAGCTCTTCAGATCGTGACCG

Total lenght is 50 nucleotides. BLASTn analysis within NCBI database reveal similarity with:

a) Vitis vinifera, whole genome shotgun sequence, contig VV78X228651.18, clone ENTAV 115

b) PREDICTED: Vitis vinifera salicylate carboxymethyltransferase (LOC100241069), mRNA

Sequence No.23

CATTTTGGTGCCCTAGAAATCRAATGAGCAAAGGTATTGACACAGTCAATGATTATTCCAAATTTTCAGGCATTTCCAAGATCAGCCACTTTAAAATACTTTTTCTTTTTTTTGGTACATAATAACAACTGAAAAAAAAAAAGAAAAAACAAAAGTTTTTTTTCTTTTTTTTGGGTACAT

Total lenght is 180 nucleotides. BLASTn analysis within NCBI database reveal similarity with:

a)Vitis vinifera contig VV78X112934.8, whole genome shotgun sequence

b) Vitis vinifera contig VV78X160535.4, whole genome shotgun sequence

Sequence No. 24 CTTTTGGCACAAAAATGTTGGCTCCAAACTAGGCTCTTTTAGGTATGGTATTAAATATCAATATTCTGAAATGGGATCACATAAATCAATGTGCGCTTGACCG

Total lenght is 103 nucleotides. BLASTn analysis within NCBI database reveal similarity with:

a)Vitis vinifera contig VV78X223690.5, whole genome shotgun sequence

b)Vitis vinifera contig VV78X273964.38, whole genome shotgun sequence

Sequence No.25

ACTCATTCACCAGGGCCAAAGAAACAAAGATATTTCAGTGTTRTGACATGAAATTAAACTTCAATGTGGTTTACAAACTTGAAAAAAGTTATAGTAAAAATTACAGCATTGACAGAACAAACAAAGGTGCCTCTTTTTTATGTGCTGCAAGTACTCTTATATGGTACTGTATCTGCCTG

Total lenght is 179 nucleotides. BLASTn analysis within NCBI database reveal similarity with:

a)Vitis vinifera contig VV78X045696.56, whole genome shotgun sequence

b)Vitis vinifera contig VV78X267462.3, whole genome shotgun sequence

Sequence No.26

TTCACATCCTGATTGTGTTGTTCAGCGCTCTGCACGTGTGTTGTGACCGG

Total lenght is 50 nucleotides. BLASTn analysis within NCBI database reveal similarity with:

a)Vitis vinifera contig VV78X248222.5, whole genome shotgun sequence

b)PREDICTED: Vitis vinifera phenylalanine ammonia-lyase-like (LOC100255939), mRNA

Sequence No.27

GCCCGTTGATCGAGGATTTGAACATTGCATCTTGCGGTGGTTTGCAAAAACTTCATGTTTCGGGGCTTGCTAATCTACATAGGCTTCAAGTAATTTGCTGTTATAATTTGAGAAGGATAGAAATCGATGCACCAAGCCTTCAACATCTCGTGTATCATTGTGGACGCTTG

Total lenght is 170 nucleotides. BLASTn analysis within NCBI database reveal similarity with:

a)Vitis vinifera contig VV78X271406.11, whole genome shotgun sequence

b)PREDICTED: Vitis vinifera putative F-box/LRR-repeat protein At5g02700 (LOC104880213), mRNA

Sequence No.28

CACACACCCTCCTCTGGTTACAAAACCAACTGTTGGATGAACCGCACAGGAAAGCCTCAAAATTAGGTGAACTCAGGGATTCAAACTTAACAACTGAGTCTATAACTCAAGACGGCAAGTGACTTCGATTTTTGATATTATGTCTTGGATAATGTCATTTAAACTTTGAA

Total lenght is 170 nucleotides. BLASTn analysis within NCBI database reveal similarity with:

a)Vitis vinifera, whole genome shotgun sequence, contig VV78X275140.10, clone ENTAV 115

b) PREDICTED: Vitis vinifera DNA-directed RNA polymerase II subunit 1 (LOC100264310), mRNA

Sequence No.29

ATCCGTGCCGTGTCCATTTGTGAAGCGATCTTGAAAATTTTCGAGGCGTTGCTACTGTAAATGCCTTGATCTTCTTCCATGAATT

Total lenght is 85 nucleotides. BLASTn analysis within NCBI database reveal similarity with:

a)Vitis vinifera contig VV78X144361.4, whole genome shotgun sequence

b)PREDICTED: Vitis vinifera tristetraprolin (LOC100262912), mRNA

Sequence No.30

AACACACCTTCTTGGATGTGGTCCACAACTCAAGCGCCAAGAAAGGAGAGGTGAGGGTGATAATTGAGCTGAATTTTCGAGCTGAATT

Total lenght is 88 nucleotides. BLASTn analysis within NCBI database reveal similarity with:

a)Vitis vinifera contig VV78X113235.8, whole genome shotgun sequence

b)PREDICTED: Vitis vinifera uncharacterized LOC100261530 (LOC100261530), mRNA

Sequence No.31

GAATGTGGTATTTTACGTCCCTTTCTTCTTCTTCTAAGCTGAATT

Total lenght is 45 nucleotides. BLASTn analysis within NCBI database reveal similarity with:

a)Vitis vinifera, whole genome shotgun sequence, contig VV78X205200.8, clone ENTAV 115

b)Vitis vinifera contig VV78X080219.5, whole genome shotgun sequence

Sequence No.32

AGCCTCTGTTCTTCTTTAGATCCCTTGTTTCACTCCGATAGTATCATAAATCGGGTCGATGCAGAGGAAATGAATGCATTTCCATACTTACTATTAAGTTAAGTAAGTGAAATAGATAAAACATAATCTGGATCATGACGCATCACTTATTCTACTGGATCTTACGGAGCCTGTCATGCACGACATGATTCGGGTCTGATCATAGAAAAGATTCTCTTCAAGCGAACC AGCCTATTCTGTATGGGGCTTGCGCGGTGGTTAGAA

Total lenght is 264 nucleotides. BLASTn analysis within NCBI database reveal similarity with:

a)Vitis vinifera subsp. caucasica chloroplast DNA, complete genome, cultivar: Meskhuri Mtsvane

b)Vitis vinifera subsp. caucasica chloroplast DNA, complete genome, cultivar: Saperavi

Sequence No.33

CCACCGCAAGATGCAATGTTCAAATCCTCGATCAACGGGCAACTTGATATCAGTTGCCGAATTGCTTGTTCATCACACTGGATTTGTCTTAAACACAACTTTCGTAGCGCC

Total lenght is 111 nucleotides. BLASTn analysis within NCBI database reveal similarity with:

a) Vitis vinifera contig VV78X271406.11, whole genome shotgun sequence

b) PREDICTED: Vitis vinifera putative F-box/LRR-repeat protein At5g02700

Sequence No.34

CGATTGGCGTTTATCCCCAACAAAATATCTCGAGTTTTTTTACATACAAAGGATTTACTTGTTACTAATATAGTCTAGCCTCTGTTCTTCTTTAGATCCCTTGTTTCACTCCGATAGTATCATAAATCGGGTCGATGCAGAGGAAATGAATGCATTTCCATACTTACTATTAAGTTAAGTAAGTGAAATAGATAAAACATAATCTGGATCATGACGCATCACTTATTCTACTGGATCTTACGGAGCCTGTTCATGCACGACATGATTCGGGTCTGATCATAGAAAAGATTCTCTTCAAGCGAACCAGCCTATTCTGTATGGGGCTTGCGCGGTGGTTAGAACAAAGACACATTTGGTTGTGAAT

Total lenght is 364 nucleotides. BLASTn analysis within NCBI database reveal similarity with:

a) Vitis vinifera contig VV78X083287.5, whole genome shotgun sequence

b) Vitis vinifera subsp. caucasica chloroplast DNA, complete genome, cultivar: Rkatsiteli

Sequence No.35

CCAGTGTGATGAACAAGCAATTCGGCAACTGATATCAAGTTGCCCGTTGATCGAGGATTTGAACATTGCATCTTGCGGTGGTTTGCAAAAACTTCATGTTTCGGGGCTTGCTAATCTACATAGGCTTCAAGTAATTTGCTGTTATAATTTGAGAAGGATAGAAATCGATGCACCAAGCCTTCAACATCTCGTGTATCATTGTGGACGCTTGCCGTGTGACATGGTCTTGACGCCCT

Total lenght is 236 nucleotides. BLASTn analysis within NCBI database reveal similarity with:

a) Vitis vinifera contig VV78X271406.11, whole genome shotgun sequence

b) PREDICTED: Vitis vinifera putative F-box/LRR-repeat protein At5g02700 (LOC104880213), mRNA

Sequence No.36

CGAGCAGCGGTGATGATGTTGGAGCCGGCTT

Total lenght is 35 nucleotides. BLASTn analysis within NCBI database reveal similarity with:

a)Vitis vinifera contig VV78X049617.4, whole genome shotgun sequence

b)PREDICTED: Vitis vinifera uncharacterized LOC104880968 (LOC104880968), mRNA

Sequence No.37

CCAAAGAACTTTGTCTCTGTTTCCAGGAGATAGTTCAGCAGTATAATTGAATTTGCAGTTCTCCTTCGATAAACAACTGCAATAAGAAGAGTACTAACCTACAAGAACGTTGCAATA TCCCAGTAGTTCAAATATT

Total lenght is 136 nucleotides. BLASTn analysis within NCBI database reveal similarity with:

a)Vitis vinifera contig VV78X134156.1, whole genome shotgun sequence

b)PREDICTED: Vitis vinifera methylesterase 10-like (LOC100852692), mRNA

Sequence No.38

ACTTTGTCTCTGTTTCCAGGAGATAGTTCAGCAGTATAATTGAATTTGCAGTTCTCCTTCG

Total lenght is 61 nucleotides. BLASTn analysis within NCBI database reveal similarity with:

a)Vitis vinifera contig VV78X134156.1, whole genome shotgun sequence

b)PREDICTED: Vitis vinifera methylesterase 10-like (LOC100852692), mRNA

Sequence No.39

AAGAACTTTGTCTCTGTTTCCAGGAGATAGTTCAGCAGTATAATTGAATTTGCAGTTCTCCTTCGATAAACAACTGCAATAAGAAGAGTACTAACCTACA

Total lenght is 100 nucleotides. BLASTn analysis within NCBI database reveal similarity with:

a)Vitis vinifera contig VV78X134156.1, whole genome shotgun sequence

b)PREDICTED: Vitis vinifera methylesterase 10-like (LOC100852692), mRNA

Sequence No.40

TGTCGAGGCCCAAAGAACTTTGTCTCTGTTTCCAGGAGATAGTTCAGCAGTATAATTGAATTTGCAGTTCTCCTTCGATAAACAACTGCAATAAGAAGAGTACTAACCTACA

Total lenght is 112 nucleotides. BLASTn analysis within NCBI database reveal similarity with:

a)Vitis vinifera contig VV78X134156.1, whole genome shotgun sequence

b)PREDICTED: Vitis vinifera methylesterase 10-like (LOC100852692), mRNA

Sequence No.41

TTCTTTGTCTTCAGTGGAAGGCTCATCCAAGAAGATGTCCAGAGCGCTGTCTGAGACCGATCTCCG

Total lenght is 66 nucleotides. BLASTn analysis within NCBI database reveal similarity with:

a)Vitis vinifera, whole genome shotgun sequence, contig VV78X071030.4, clone ENTAV 115

b)PREDICTED: Vitis vinifera uncharacterized LOC100257716 (LOC100257716), mRNA
