# Supplementary material for: Dynamics and Reversibility of the DNA Methylation Landscape of Grapevine Plants (Vitis vinifera) Stressed by In Vitro Cultivation and Thermotherapy
Source: PLoS One. 2015 May 14;10(5):e0126638. doi: 10.1371/journal.pone.0126638 (PMC4431845; doi:10.1371/journal.pone.0126638)
Supplement: S5 Table — (DOCX) [file pone.0126638.s006.docx]

**Table S5 - Impact of individual stressing factors on intensity of DNA methylation changes**

| Virus infection* | In vitro  cultivation  (1 year) | In vitro thermotherapy (1year) | In vitro cultivation  (8 weeks) | In vitro thermotherapy  (8 weeks) |
| --- | --- | --- | --- | --- |
| MT = 8.24 %  R = 13.74 % | MT = 23.47 %  R = 23.07 % | MT = 28.39 %  R = 32.11 % | MT = 22.0 %  R = 39.67 % | MT = 37.87 %  R = 37.84 % |

* Data shows percentage of polymorphic MSAP amplicons if individual stressed variants are compared with not-infected maternal plant
